# Efficient Two-Stage Autofocus for Micro-Assembly Based on Joint Spatial-Frequency Image Quality Assessment

**DOI:** 10.3390/jimaging12030137

**Published:** 2026-03-19

**Authors:** Jianpeng Zhang, Tianbo Kang, Xin Zhao, Mingzhu Sun, Yi Yang

**Affiliations:** 1 National Key Laboratory of Intelligent Tracking and Forecasting for Infectious Diseases, Engineering Research Center of Trusted Behavior Intelligence, Ministry of Education, Tianjin Key Laboratory of Intelligent Robotics, Institute of Robotics and Automatic Information System, Nankai University, Tianjin 300350, China; 2120240578@mail.nankai.edu.cn (J.Z.); 1120240268@mail.nankai.edu.cn (T.K.); zhaoxin@nankai.edu.cn (X.Z.); 2 Institute of Intelligence Technology and Robotic Systems, Shenzhen Research Institute of Nankai University, Shenzhen 518083, China; 3 Research Center of Laser Fusion, China Academy of Engineering Physics, Mianyang 621000, China

**Keywords:** micro-assembly, micro-vision, autofocus, no-reference image quality assessment, spatial-frequency modeling, dual-camera system

## Abstract

Reliable autofocus is a fundamental prerequisite for precise positioning in micro-assembly systems, where complex reflections, scale variations, and narrow depth-of-field often degrade the robustness of traditional sharpness metrics. To address these challenges, we propose an efficient two-stage autofocus method for a dual-camera micro-vision system based on a spatial-frequency image quality assessment (IQA) model. First, we design WaveMamba-IQA for image sharpness estimation, synergistically combining the Discrete Wavelet Transform with Vision Transformers to capture high-frequency details and semantic features, further enhanced by Multi-Linear Transposed Attention and Vision Mamba for global context modeling. Moreover, we implement a coarse-to-fine autofocus workflow, employing the Covariance Matrix Adaptation Evolution Strategy for global optimization on the horizontal camera, followed by geometric prior-based precise adjustment for the oblique camera. Experimental results on a custom microsphere dataset demonstrate that WaveMamba-IQA achieves a Spearman correlation coefficient of 0.9786. Furthermore, the integrated system achieves a 98.33% autofocus success rate across varying lighting conditions. This method significantly improves the robustness and automation level of micro-assembly systems, effectively overcoming the limitations of manual and traditional focusing techniques.

## 1. Introduction

Micro-assembly technology is a critical capability for performing micrometer-level precision operations and has been widely applied in fields such as aerospace, biomedicine, and micro-electromechanical systems [1,2]. As the miniaturization and complexity of devices demand higher precision and automation, integrated micro-vision systems have become the key means to guide micro-assembly robots in achieving precise operations [3,4]. Among numerous micro-assembly applications, the precision assembly of microspheres and microtubes is a typical yet highly challenging task [5]. This process imposes strict requirements on the accurate estimation of the microsphere’s spatial position, a prerequisite that heavily relies on the micro-vision system’s ability to capture the microsphere profile clearly [6]. However, in actual operating environments, complex reflection interference and variations in microsphere size often lead to unstable performance of traditional autofocus strategies [7]. Consequently, obtaining reliable and clear imaging results is difficult, forcing current assembly processes to rely largely on manual focusing. This severely constrains improvements in system efficiency and full automation [8]. Therefore, investigating autofocus methods for micro-assembly vision systems is of important practical significance. Reliable autofocus can provide clear image information for microsphere position estimation and precise assembly, while also reducing the dependence on manual operation and improving the efficiency and automation level of the micro-assembly process.

The core of building a robust autofocus system lies in accurate image sharpness evaluation. Traditional sharpness evaluation functions based on mathematical statistics and gradient information have long been widely used in autofocus applications due to their simplicity [9,10]. For instance, the Tenengrad gradient operator has been extensively adopted as a focus measure and successfully applied to autofocus tasks in microsphere assembly [11]. In addition, grayscale statistical functions such as variance, as well as edge-based methods including the Brenner gradient and Laplacian energy, are also commonly used in autofocus systems [12]. These methods mainly quantify image sharpness through high-frequency responses or statistical variations. However, because they rely on hand-crafted features, their performance is easily affected by noise, illumination changes, and background interference, resulting in limited robustness in complex micro-assembly environments.

To overcome these limitations, recent studies have begun to explore learning-based sharpness assessment for autofocus and image quality evaluation. For example, alternative formulations such as the Logarithmic Image Processing (LIP) framework have been introduced to improve robustness under illumination fluctuations [13], and Kolmogorov–Arnold networks (KAN) have been investigated for image sharpness assessment with improved generalization ability [14]. These advances suggest that data-driven methods can provide a more flexible and robust solution than traditional hand-crafted focus measures.

Meanwhile, advances in no-reference image quality assessment (NR-IQA) have provided a promising direction for autofocus in micro-assembly systems [15,16,17]. Deep learning-based NR-IQA models, such as MANIQA, HyperIQA, and MetaIQA [18,19,20,21], learn deep semantic and structural representations directly from images and have achieved substantially better performance than traditional sharpness operators in general image quality assessment tasks. This indicates their potential to improve autofocus robustness under noise and environmental variations [22].

Nevertheless, existing sharpness assessment and IQA methods remain insufficient for micro-assembly autofocus. On the one hand, most existing methods are developed for general image quality assessment or natural image sharpness evaluation, rather than for micrometer-level autofocus tasks in micro-assembly scenarios. On the other hand, they are often insufficiently sensitive to subtle defocus variations caused by slight camera displacement, especially under strong specular reflections, background interference, and target size variability. Furthermore, many existing architectures mainly focus on spatial-domain representation learning while underutilizing frequency-domain information that is crucial for discriminating fine focus differences [23,24,25]. As a result, existing methods still struggle to provide reliable sharpness evaluation for precise microsphere assembly.

To address these challenges, this study presents an efficient two-stage autofocus framework for micro-assembly based on a spatial-frequency IQA model termed WaveMamba-IQA, coupled with geometry-guided dual-camera constraints. First, we construct a joint spatial-frequency IQA model that integrates the Discrete Wavelet Transform (DWT) [26] with a Vision Transformer (ViT) [27] to exploit complementary high-frequency detail and spatial semantic features for autofocus. Within the model, Multi-Linear Transposed Attention (MLTA) [18,28] is introduced to model global channel dependencies, and a Vision Mamba state space module [29] is adopted to capture global context with linear complexity for robust sharpness scoring. Second, leveraging the geometric constraints of the dual-camera micro-vision system, which comprises a global horizontal camera and a high-magnification oblique camera, we design a practical autofocus workflow. During the focus search stage, the Covariance Matrix Adaptation Evolution Strategy (CMA-ES) [30] is employed to mitigate local extrema that commonly hinder traditional quadratic fitting or hill-climbing methods.

Our contributions are summarized as follows.

1. We propose WaveMamba-IQA, a joint spatial-frequency IQA model for autofocus that integrates DWT and ViT for complementary frequency and spatial feature modeling, and further combines MLTA with a Vision Mamba state space module to enable robust sharpness scoring.
2. We employ CMA-ES in the focus search stage to alleviate local-extrema issues encountered by conventional quadratic fitting or hill-climbing strategies, improving robustness in reflection-dominated scenes.
3. We design a geometry-constrained dual-camera autofocus workflow that couples a global horizontal camera with a high-magnification oblique camera for efficient initialization and refinement.

The remainder of this paper is organized as follows. Section 2 introduces the proposed autofocus method. Section 3 describes the experimental setup and implementation details. Section 4 and Section 5 present the experimental results and discussion. Finally, Section 6 concludes this paper.

## 2. Method

### 2.1. System Setup

**Figure 1:**
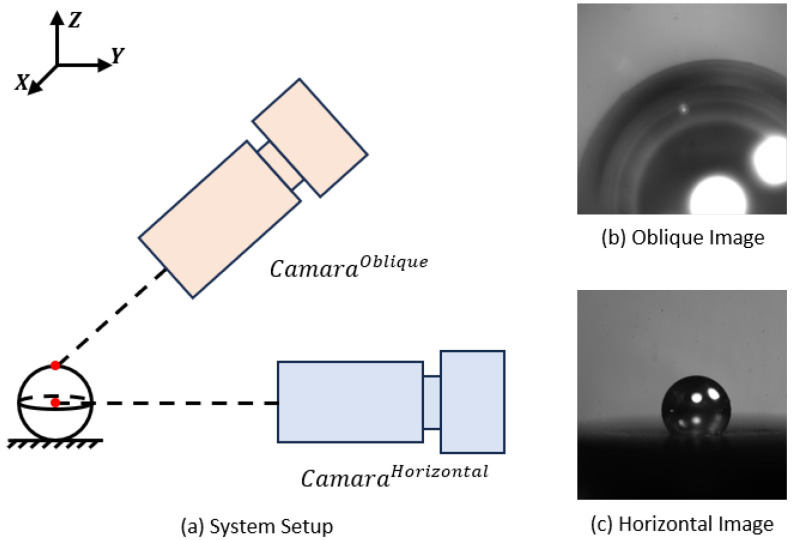
Dual-Camera Micro-Vision System. (**a**) System configuration. (**b**) Oblique-view microsphere image. (**c**) Horizontal-view microsphere image.

As shown in Figure 1, the system employs a dual-camera micro-vision architecture. The horizontal camera has a lower magnification and is primarily used for global observation; the oblique camera features high magnification, capable of capturing high-resolution microsphere edges. The focusing motion is realized by a motor-controlled robotic manipulator that clamps and moves the camera, providing a minimum incremental motion of 1 µm and a bidirectional repeatability of ±2 µm (N=20), measured over *N* repeated approaches to the same target position. Both cameras use the MV-CH250-60TM-M58S-NF model. The horizontal optical setup provides a magnification range of 1.36×–10× with a depth of field of 0.89–0.03 mm and an NA of 0.08, while the oblique optical setup provides a magnification range of 2.04×–15× with a depth of field of 0.4–0.01 mm and an NA of 0.12.

By achieving clear imaging and fitting the microsphere center in the oblique field of view, and combining this information with camera calibration parameters, the system provides accurate geometric cues for subsequent spatial localization, thereby satisfying the stringent positioning accuracy requirements of the microsphere and microtube assembly.

### 2.2. Overall Pipeline

**Figure 2:**
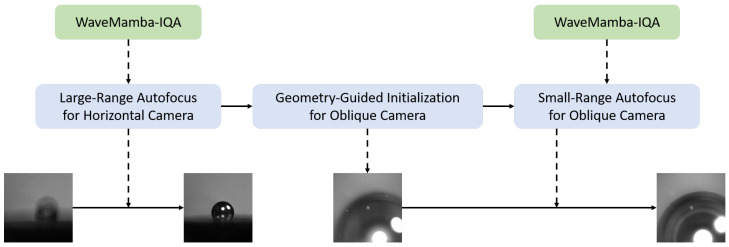
Overall Pipeline of the Proposed Dual-Camera Autofocus Method.

The overall process of this method is shown in Figure 2. It primarily consists of four key modules:

- WaveMamba-IQA sharpness evaluation model;
- Large-range autofocus for the horizontal camera;
- Calculation of the initial position for the oblique camera based on geometric priors;
- Small-range fine autofocus for the oblique camera.

During the focusing process, images acquired by the horizontal camera are input into the WaveMamba-IQA model for no-reference sharpness scoring. By jointly modeling the spatial and frequency domain features of the image, the model calculates reliable sharpness scores for images at different positions. On this basis, the horizontal camera employs the CMA-ES to perform global optimization within a large search space to accurately locate the optimal position. Subsequently, the theoretical clear position of the oblique camera is derived by leveraging known prior information on microsphere size together with the geometric relationship between the two cameras. However, due to the existence of assembly errors and microsphere manufacturing tolerances, a small-range search is still required within its neighborhood, thereby achieving high-precision autofocus for both cameras throughout the process.

### 2.3. WaveMamba-IQA Model

#### 2.3.1. Overall Architecture

**Figure 3:**
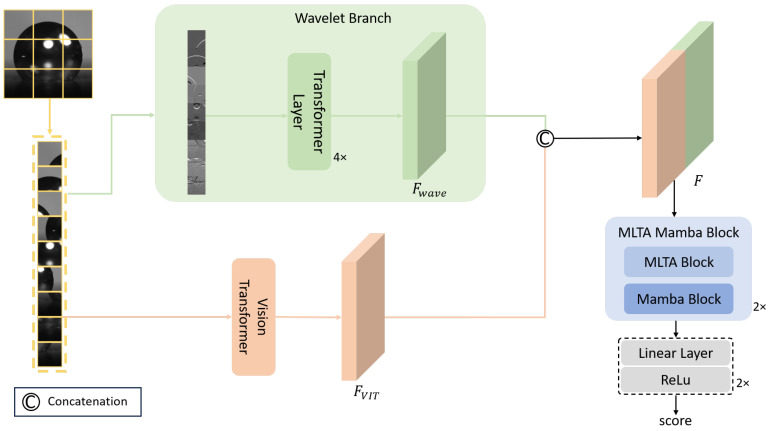
WaveMamba-IQA Architecture with Parallel Wavelet–ViT Feature Extraction and MLTA Mamba Fusion.

WaveMamba-IQA is a joint spatial-frequency no-reference image quality assessment model designed for autofocus tasks. Its overall network structure is shown in Figure 3. Given an input image $I\in R^{H\times W\times 3}$, the model first divides it into *N* non-overlapping patches, denoted as ${\left\{x_{i}\right\}}_{i=1}^{N}$. Subsequently, all patches are fed in parallel into two complementary feature extraction branches: one modeling frequency-domain detail information and the other capturing spatial semantic information closely related to image sharpness.

On one hand, the wavelet branch performs multi-scale frequency decomposition on each patch via DWT [26] and combines it with Transformer layers to model high-frequency sub-bands. Here, *P* denotes the patch size, and the token grid resolution is $\frac{H}{P}\times \frac{W}{P}$. This effectively captures edge and texture detail features highly correlated with image sharpness, yielding the frequency-domain feature representation $F_{\mathrm{wave}}\in R^{\frac{H}{P}\times \frac{W}{P}\times C_{\mathrm{wave}}}$ [31].

On the other hand, let $F_{i}\in R^{\frac{H}{P}\times \frac{W}{P}\times C_{i}}$ denote the feature map from the $i^{\mathrm{th}}$ layer of the Vision Transformer. To enhance the model’s ability to represent information at different receptive field scales, this study concatenates the intermediate features from the 6th to the 9th layers along the channel dimension to form the spatial semantic feature representation $F_{\mathrm{ViT}}\in R^{\frac{H}{P}\times \frac{W}{P}\times \sum_{i=6}^{9}C_{i}}$.

Subsequently, features from both branches are concatenated in the channel dimension to obtain the joint feature representation:

(1) $$F=\mathrm{Concat}\left(F_{\mathrm{wave}},\;F_{\mathrm{ViT}}\right),\;F\in R^{\frac{H}{P}\times \frac{W}{P}\times \left(C_{\mathrm{wave}}+\sum_{i=6}^{9}C_{i}\right)}$$

The fused feature *F* is further fed into the MLTA Mamba Block for global modeling to enhance the capability of modeling long-range dependencies. Finally, the image sharpness score is regressed through a linear layer followed by a ReLU activation function, and is used for sharpness evaluation in the autofocus task.

#### 2.3.2. Wavelet Branch

In the wavelet branch, the model first performs DWT [26] on each input patch, decomposing it into one low-frequency sub-band and multiple high-frequency sub-bands, thereby explicitly separating the structural information from detail information. Let an input patch be denoted as *x*; its wavelet decomposition can be formally expressed as:

(2) $$\mathrm{DWT}\left(x\right)=\left\{x_{\mathrm{LL}},\;x_{\mathrm{LH}},\;x_{\mathrm{HL}},\;x_{\mathrm{HH}}\right\}$$

Here, $x_{\mathrm{LL}}$ represents the low-frequency approximation component, mainly containing the overall brightness and structural information of the image; whereas $x_{\mathrm{LH}}$, $x_{\mathrm{HL}}$, and $x_{\mathrm{HH}}$ represent high-frequency detail components in different directions, concentrating on edges, textures, and subtle structural changes. Subsequently, all low-frequency and high-frequency sub-bands are concatenated in the channel dimension and uniformly fed into Transformer layers for feature modeling. This process allows for learning global dependencies among different frequency components while preserving multi-scale frequency information, thereby extracting a more discriminative frequency domain feature representation. Compared to NR-IQA models like MANIQA, HyperIQA, and MetaIQA which rely primarily on spatial features [18,19,20], the introduction of explicit frequency domain modeling significantly enhances the model’s perceptual ability regarding edge blur and high-frequency detail degradation. This characteristic makes the frequency domain branch more aligned with the requirements of autofocus tasks in analyzing imaging sharpness. Experiments demonstrate that this wavelet branch effectively improves model performance.

#### 2.3.3. MLTA Mamba Block

To achieve efficient fusion of spatial and frequency domain features, we introduce the MLTA Mamba Block, which consists of two core units: the MLTA Block and the Vision Mamba Block. In our framework, both branches are aligned on the same token grid resolution $\frac{H}{P}\times \frac{W}{P}$. The frequency branch applies DWT to each patch and encodes the resulting sub-band responses into the channel dimension, yielding $F_{\mathrm{wave}}\in R^{\frac{H}{P}\times \frac{W}{P}\times C_{\mathrm{wave}}}$, while the ViT branch extracts spatial semantic features on the same token grid. The fused representation *F* is obtained by channel-wise concatenation as defined in Equation (1).

**Figure 4:**
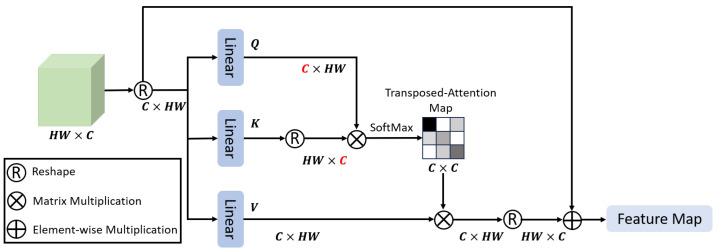
MLTA Block with Token-Grid-Channel Dimension Exchange for Channel-Wise Attention.

The architecture of the MLTA Block is shown in Figure 4. Instead of computing attention over the token-grid dimension, MLTA performs self-attention along the channel dimension by exchanging the token-grid and channel axes. Under the above tensor layout, channel-wise attention directly models global correlations among channels, including correlations between different wavelet sub-bands as well as cross-domain interactions between wavelet-derived frequency channels and ViT-derived semantic channels. In this way, MLTA serves as a lightweight fusion operator that preserves frequency-domain cues while selectively enhancing spatial-frequency complementarity.

This transposed attention mechanism can implicitly encode global context information while significantly reducing computational complexity, efficiently enhancing the fused features from the spatial and frequency branches [28].

**Figure 5:**
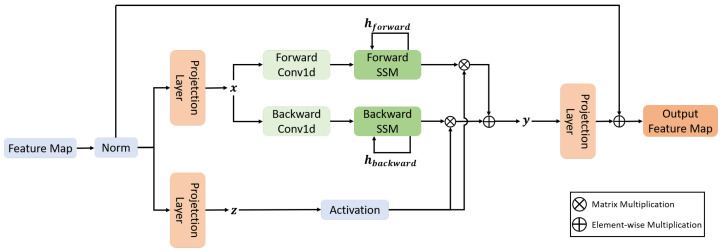
Vision Mamba Block with Bidirectional State Space Modeling.

Mamba, based on the State Space Model (SSM), has demonstrated the ability to efficiently capture long-range dependencies with linear complexity in one-dimensional long-sequence modeling tasks [32]. However, its original structure is mainly targeted at 1D sequences and is difficult to directly adapt to the modeling needs of 2D feature structures in visual tasks. To this end, Vision Mamba [29] proposed the Vim Block. As explicitly illustrated in Figure 5, the 2D feature map is first reshaped into a 1D token sequence, and a bidirectional design is adopted to model long-range dependencies by processing the sequence in both forward and reverse orders. Concretely, a reverse-order branch performs the same local token mixing and SSM-based sequence scanning on the reversed sequence, and its output is then restored to the original order and fused with the forward-branch output by averaging, enabling the block to aggregate contextual information from both directions and thereby capture long-range dependencies in the reshaped token sequence with linear complexity. This paper incorporates the Vim Block into the WaveMamba-IQA framework to perform efficient global modeling on the spatial-frequency fused features, thereby improving sharpness assessment accuracy and robustness in autofocus tasks while maintaining linear computational complexity.

### 2.4. Autofocus Procedure

#### 2.4.1. Large-Range Global Autofocus for the Horizontal Camera

In practical application scenarios, the microsphere is placed on the surface of a holder. However, the specific position of the microsphere varies with each placement. This positional inconsistency necessitates re-executing the full autofocus procedure after replacing the microsphere. In the first stage of autofocus, the system performs large-range focusing for the horizontal camera to quickly determine the camera’s optimal position. Let the camera position along the optical axis be *w*, and the image acquired at different *w* be denoted as I(w). The WaveMamba-IQA model is used as the sharpness evaluation function, and its output score can be expressed as:

(3) $$Q\left(w\right)=f_{\mathrm{WaveMamba}}\left(I(w)\right)$$

where $f_{\mathrm{WaveMamba}}\left(\cdot \right)$ represents the proposed spatial-frequency IQA model. To mitigate noise sensitivity and local-extrema convergence in conventional autofocus strategies, this paper employs CMA-ES for global optimization of the camera position. CMA-ES iteratively samples candidate solutions near the mean. By evaluating the sharpness of images at candidate positions using the WaveMamba-IQA model to obtain fitness scores, it adaptively updates the mean and covariance of the search distribution. This allows for efficiently approximating the global optimal solution with fewer sampling iterations. The optimization process can be formulated as:

(4) $$\left(\mu_{t+1},\;C_{t+1}\right)=\mathrm{CMA}-\mathrm{ES}\left(\mu_{t},\;C_{t},\;\left\{Q\left(w\right)|w∼N\left(\mu_{t},C_{t}\right)\right\}\right)$$

(5) $$w_{t}^{*}=\underset{w∼N(\mu_{t},C_{t})}{\mathrm{argmax}}Q\left(w\right)$$

Here, $\mu_{t}$ represents the mean of the search distribution of CMA-ES at the $t^{\mathrm{th}}$ iteration, while $C_{t}$ denotes the corresponding covariance matrix. Together, they characterize the center position and distribution shape of candidate solutions in the current camera position search space. The symbol $w∼N(\mu_{t},C_{t})$ denotes random sampling of camera positions under this Gaussian distribution to generate a set of candidate positions. For each candidate position *w*, the corresponding image is acquired and input into the WaveMamba-IQA model to obtain the sharpness score Q(w), which serves as the fitness function for CMA-ES. In the $t^{\mathrm{th}}$ iteration, $w_{t}^{*}$ represents the candidate camera position with the highest sharpness score in the current round. Based on the scores of all candidate solutions, CMA-ES adaptively updates the search distribution mean $\mu_{t+1}$ and covariance matrix $C_{t+1}$ in the ${\left(t+1\right)}^{\mathrm{th}}$ iteration, causing the search distribution to gradually converge towards the high-quality imaging region. When the optimal score does not improve for three consecutive generations, the optimization process is considered converged, and the corresponding $w^{*}$ is taken as the final optimal clear imaging position.

#### 2.4.2. Oblique Camera Initial Position Estimation Based on Geometric Priors

After completing the horizontal camera focusing, the system further focuses the oblique camera. Considering that in this micro-assembly system, there is a fixed geometric relationship between the horizontal and oblique cameras when both are in a focused state, we can use the microsphere size information and the change in the horizontal camera’s position to estimate the prior clear imaging position of the oblique camera.

**Figure 6:**
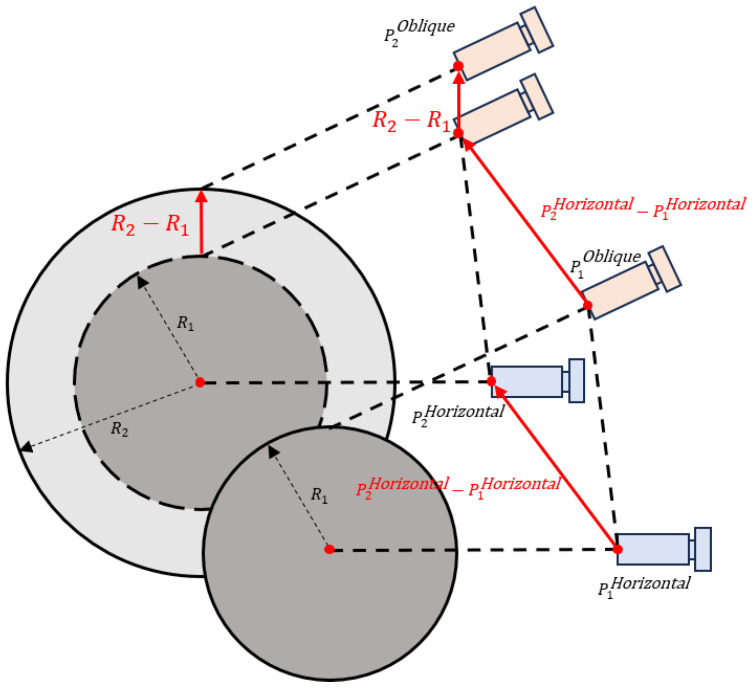
Schematic of the Geometric Relationship Between Microsphere Radius and Camera Positions.

As shown in Figure 6, assume that when the microsphere radius is $R_{1}$ µm, both the horizontal and oblique cameras achieve clear imaging, with the corresponding clear position of the oblique camera denoted as $P_{1}^{\mathrm{Oblique}}$ and the horizontal camera position as $P_{1}^{\mathrm{Horizontal}}$. When the actual radius of the microsphere changes to $R_{2}$ µm, the corresponding clear position of the oblique camera is denoted as $P_{2}^{\mathrm{Oblique}}$, and the horizontal camera position is denoted as $P_{2}^{\mathrm{Horizontal}}$. Under ideal geometric conditions, a change in microsphere radius will cause a displacement of the apex of the microsphere. Simultaneously, due to different placement positions of the microsphere, the horizontal camera’s position will also change accordingly during refocusing. Comprehensively considering these two factors, the clear imaging position of the oblique camera $P_{2}^{\mathrm{Oblique}}$ can be approximated as:

(6) $$\begin{array}{cc} \Delta P^{\mathrm{Horizontal}} & =P_{2}^{\mathrm{Horizontal}}-P_{1}^{\mathrm{Horizontal}}, \end{array}$$

(7) $$\begin{array}{cc} \Delta R & =R_{2}-R_{1}, \end{array}$$

(8) $$\begin{array}{cc} P_{2}^{\mathrm{Oblique}} & =P_{1}^{\mathrm{Oblique}}+\Delta P^{\mathrm{Horizontal}}+\Delta R\;e_{z} \end{array}$$

where $\Delta P^{\mathrm{Horizontal}}$ denotes the displacement of the horizontal camera during refocusing, ΔR denotes the microsphere radius variation and $e_{z}$ represents the unit vector along the camera optical axis direction.

The above geometric relationship provides a reasonable and interpretable initial clear position estimate for the oblique camera, effectively reducing the search space for subsequent focusing and improving the efficiency of autofocus. In the actual micro-assembly process, uncertainties such as assembly errors, microsphere manufacturing tolerances, and system calibration errors inevitably introduce deviations from the ideal geometric model. Nevertheless, these errors are relatively small, and the estimated position $P_{2}^{\mathrm{Oblique}}$ remains sufficiently close to the true focal position. Therefore, a small-range fine focusing search around this initial estimate is sufficient to further enhance the imaging sharpness and focusing accuracy of the oblique camera.

#### 2.4.3. Small-Range Fine Autofocus for the Oblique Camera

In the small-range fine autofocus for the oblique camera, a localized search is performed centered on the estimated oblique camera clear position $P_{2}^{\mathrm{Oblique}}$. Specifically, a set of candidate images is acquired within its neighborhood along the camera optical axis with a fixed step size Δw. The set of positions can be expressed as:

(9) $$P=\left\{\;P_{2}^{\mathrm{Oblique}}+k\;\Delta w\;|\;k\in \left\{-K,-K+1,\ldots ,K\right\}\;\right\}$$

where *K* represents the number of steps corresponding to the search radius, determining the range of the local search interval. All candidate images are input into the WaveMamba-IQA model for sharpness scoring, and the optimal offset index $k^{*}$ is selected for the oblique camera. The optimal clear imaging position $P^{*}$ is then calculated from $k^{*}$, the initial position $P_{2}^{\mathrm{Oblique}}$ and the step increment Δw:

(10) $$k^{*}=\underset{k\in [-K,K]}{\mathrm{argmax}}f_{\mathrm{WaveMamba}}\left(I\left(P_{2}^{\mathrm{Oblique}}+k\Delta w\right)\right)$$

where the optimal clear imaging position is derived as:

(11) $$P^{*}=P_{2}^{\mathrm{Oblique}}+k^{*}\Delta w$$

Since the search range in this stage is effectively constrained by the geometric prior estimation from the previous stage, the focusing process only needs to be conducted within a limited local interval. This allows for high-precision autofocus with very few image sampling iterations, effectively balancing the system’s focusing efficiency and robustness.

The overall autofocus procedure is summarized in Algorithm 1.
**Algorithm 1** Two-Stage Autofocus Procedure.**Require:** $P_{1}^{\mathrm{Horizontal}}$, $P_{1}^{\mathrm{Oblique}}$, $R_{1}$, $R_{2}$, Δw, *K***Ensure:** $w^{*}$, $P^{*}$1:Use CMA-ES and WaveMamba-IQA to search the optimal horizontal camera position $w^{*}$.2:Set $P_{2}^{\mathrm{Horizontal}}\leftarrow w^{*}$3:Compute $\Delta P^{\mathrm{Horizontal}}=P_{2}^{\mathrm{Horizontal}}-P_{1}^{\mathrm{Horizontal}}$ and $\Delta R=R_{2}-R_{1}$4:Estimate $P_{2}^{\mathrm{Oblique}}=P_{1}^{\mathrm{Oblique}}+\Delta P^{\mathrm{Horizontal}}+\Delta R\;e_{z}$5:**for** k∈{−K,−K+1,…,K} **do**6:    Evaluate $f_{\mathrm{WaveMamba}}\left(I\left(P_{2}^{\mathrm{Oblique}}+k\Delta w\right)\right)$7:**end for**8:Compute $k^{*}={\mathrm{argmax}}_{k\in \{-K,\ldots ,K\}}f_{\mathrm{WaveMamba}}\left(I\left(P_{2}^{\mathrm{Oblique}}+k\Delta w\right)\right)$9:Set $P^{*}=P_{2}^{\mathrm{Oblique}}+k^{*}\Delta w$10:**return** $w^{*}$, $P^{*}$

## 3. Experiments

### 3.1. Datasets

This study constructed a microscopic image dataset for microsphere autofocus experiments, and the detailed dataset statistics were summarized in Table 1. All images were acquired on a real micro-assembly experimental platform using the global horizontal camera and the high-magnification oblique camera integrated into the system.

**Figure 7:**
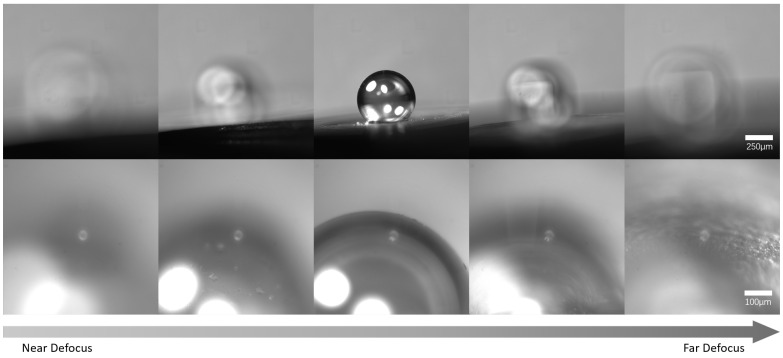
Representative Image Sequences Showing Sharpness Variation from Near Defocus to Far Defocus.

The data acquisition process employed a systematic scanning strategy to construct a comprehensive dataset. We controlled the camera to move in fixed steps along its optical axis, capturing the image of the current field of view at each interval. The acquired image sequences completely cover the imaging transition from Near Defocus through Clear to Far Defocus. This acquisition method ensures that the dataset encompasses a wide spectrum of sharpness states, ranging from severe blur to fine texture, thereby enabling the model to effectively learn sensitivity to camera position changes. Furthermore, the dataset covers scenarios with different lighting conditions and microspheres of different sizes, reflecting challenges such as reflection interference and target scale differences present in actual assembly. Figure 7 displays images from a typical acquired sequence.

Regarding image labeling, this study used the distance between the camera position corresponding to each image and the clear imaging position in that sequence as the basis. This distance was normalized to the [0,1] interval via linear mapping to serve as the image sharpness label. The clear imaging position in each sequence was determined by manual visual inspection, selecting the image that appeared sharpest to the human eye. The score corresponding to the clearest imaging position was set to 1, and the image score decreased correspondingly as the camera position gradually deviated from the focal point.

### 3.2. Implementation Details

Our experiments were implemented on an NVIDIA GeForce RTX 4090 GPU with PyTorch 2.2.0 and CUDA 11.8 for training and testing.

In the model initialization phase, ViT-B/8 [27] pretrained on the ImageNet-21k dataset and fine-tuned on ImageNet-1k was selected as the initial weights for the backbone network, with the patch size set to 8. Following the standard ViT-B configuration, the backbone uses an embedding dimension of 768 with 12 Transformer blocks and 12 attention heads, and employs learnable absolute positional embeddings. During training, the batch size was set to 16 to maximize GPU memory utilization, and the ViT-B/8 backbone was fine-tuned end-to-end. Given that Vision Transformer and its variants typically required fixed-size inputs, while the original acquired image resolution was 512 × 512, uniform preprocessing was performed on the data during the model input phase.

In the model training phase, to improve the model’s generalization ability and mitigate overfitting, image patches of size 224 × 224 were randomly cropped from the original training images as network input. Additionally, random horizontal flipping was performed on the cropped images with a probability of 0.5 to enhance the diversity of training samples. During model prediction, to improve the stability of sharpness prediction results, a multi-view averaging strategy was adopted. Specifically, 10 image patches of size 224 × 224 were randomly cropped from each test image and individually input into the model for score prediction. The average of these 10 predictions was taken as the final sharpness score for that image.

For the wavelet branch, the wavelet tokens are projected to an embedding dimension of 384 and processed by a lightweight Transformer encoder consisting of 4 Transformer layers with 8 attention heads per layer and an MLP expansion ratio of 2.0. For the ViT branch, we extract intermediate patch-token features from the 6th to the 9th Transformer blocks, discard the class token, and concatenate the remaining patch tokens along the channel dimension in a token-wise manner, yielding a 4×768 dimensional spatial feature. The extracted features are taken directly from each block output and concatenated without additional pooling.

The Adam optimizer was used with a weight decay of $1\times 10^{-5}$. A cosine annealing learning rate schedule was adopted, with the maximum iteration period $T_{\max}$ set to 50 and the minimum learning rate $\eta_{\min}$ set to 0. During model training, the Mean Squared Error (MSE) loss function was used to minimize the difference between the model’s predicted sharpness score and the manually annotated score.

To ensure the reliability and reproducibility of the experimental results, the dataset was randomly divided into training and testing sets at an 8:2 ratio at the sequence level, where one image sequence was selected as the test set and the remaining sequences were used for training. All experiments were repeated with five different random seeds, and across these runs, different image sequences were used as the test set, such that all four sequences were covered at least once. The final results were reported as the average values of all evaluation metrics over the five runs.

### 3.3. Evaluation Metrics

This paper adopted the Spearman’s Rank-Order Correlation Coefficient (SROCC) and Pearson’s Linear Correlation Coefficient (PLCC) as evaluation metrics to quantitatively assess the performance of the proposed method. Both metrics were calculated based on the relative relationships among scores of multiple images, effectively characterizing the sorting consistency and trend correlation of sharpness with camera position changes. This was more aligned with the practical requirement of autofocus tasks to determine direction based on score changes.

PLCC was used to measure the linear correlation between the model’s predicted sharpness scores and the ground truth scores, defined as follows:

(12) $$\mathrm{PLCC}=\frac{\sum_{i=1}^{N}\left(s_{i}-\mu_{s}\right)\left({\hat{s}}_{i}-{\hat{\mu }}_{s}\right)}{\sqrt{\sum_{i=1}^{N}{\left(s_{i}-\mu_{s}\right)}^{2}\sum_{i=1}^{N}{\left({\hat{s}}_{i}-{\hat{\mu }}_{s}\right)}^{2}}}$$

where $s_{i}$ and ${\hat{s}}_{i}$ represent the ground truth sharpness score and the model predicted score for the $i^{\mathrm{th}}$ test image, respectively; $\mu_{s}$ and ${\hat{\mu }}_{s}$ represent the means of the ground truth scores and predicted scores, respectively; and *N* represents the number of test images.

SROCC was used to measure the consistency between the predicted scores and ground truth scores at the ranking level. Let $d_{i}$ denote the difference in rank between the ground truth score and the predicted score for the $i^{\mathrm{th}}$ test image; the definition of SROCC is:

(13) $$\mathrm{SROCC}=1-\frac{6\sum_{i=1}^{N}d_{i}^{2}}{N(N^{2}-1)}$$

The value range for both PLCC and SROCC is [−1,1]. A larger value indicates a stronger correlation and consistency between the model prediction results and the true scores, indicating superior image sharpness evaluation performance of the model.

## 4. Results

### 4.1. WaveMamba-IQA Model Performance

To verify the effectiveness of the proposed method, this paper conducted comparative experiments between WaveMamba-IQA and current representative No-Reference Image Quality Assessment methods, MANIQA [18] and HyperIQA [19]. All methods were trained under the same data split and training strategy. Table 2 reports the mean ± standard deviation over five random seeds.

In addition, paired two-sided *t*-tests were conducted to further assess whether the improvements of WaveMamba-IQA over the competing methods were statistically significant, and the corresponding *p*-values are reported in Table 3.

As can be seen from the Table 2, WaveMamba-IQA achieved the best performance in both SROCC and PLCC metrics under both the horizontal and oblique camera views. In the horizontal view, the SROCC and PLCC of WaveMamba-IQA reached 0.9786 and 0.9624, respectively, improving by 0.82% and 0.35% percentage points compared to MANIQA [18]. In the oblique view, it also outperformed the comparison methods. This indicates that the proposed method possesses more stable and precise sharpness modeling capabilities under different magnifications. Furthermore, the paired two-sided *t*-test results in Table 3 show that the proposed method achieves statistically significant improvements on most metrics. In particular, the gains on SROCC are especially meaningful for autofocus, since SROCC mainly reflects the relative ranking consistency of image sharpness scores and is therefore more closely aligned with the objective of identifying the clearest focal position.

**Figure 8:**
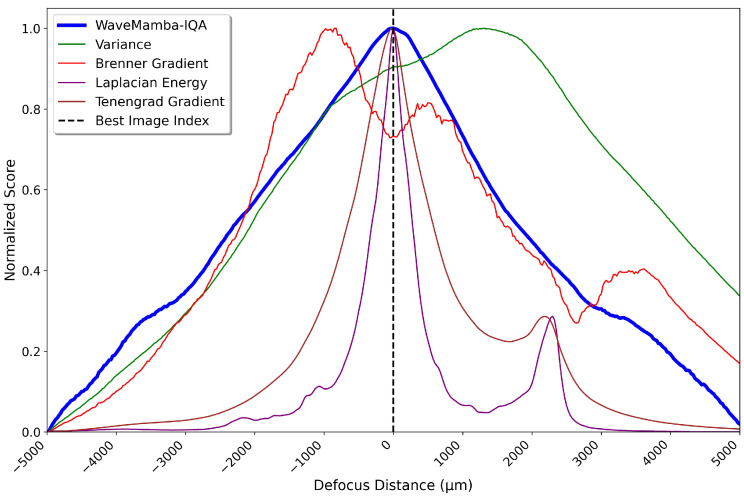
Comparison of Sharpness Curves Between WaveMamba-IQA and Traditional Metrics.

In addition, to benchmark the proposed method against traditional sharpness evaluation functions, we collected 10 sets of horizontal microsphere image sequences under varying illumination conditions, covering the full range from Near Defocus through Clear to Far Defocus. From these, one sequence was randomly selected for evaluation. We calculated sharpness scores using multiple traditional sharpness functions, including Variance, Brenner Energy, Laplacian Energy, and Tenengrad Gradient [12], as well as the proposed WaveMamba-IQA model, applying normalization to the results of all methods. The comparative results are illustrated in Figure 8. Compared with traditional methods, the scoring curve generated by WaveMamba-IQA exhibits superior monotonicity and stability with respect to changes in the camera’s axial position, thereby significantly facilitating the search for the optimal focal position during the autofocus process.

### 4.2. Ablation Study Analysis

To further verify the contribution of the wavelet branch in WaveMamba-IQA, we conducted an ablation study under the same training and testing configuration. Specifically, besides the full WaveMamba-IQA, we considered a simplified variant without the wavelet branch, denoted as Mamba (w/o Wavelet), and another variant in which the DWT operation in the wavelet branch was replaced with a Discrete Fourier Transform (DFT) [33] operation while keeping the rest of the branch unchanged, denoted as Mamba (w/ DFT). The comparison results are reported in Table 4.

In addition, to examine whether the observed performance gains are statistically significant, we further conducted paired two-sided *t*-tests between WaveMamba-IQA and the two ablation variants. The corresponding *p*-values are summarized in Table 5, where p<0.05 indicates a statistically significant difference.

As shown in Table 4, the complete WaveMamba-IQA consistently achieved the best performance across all four evaluation metrics. Compared with Mamba (w/o Wavelet), WaveMamba-IQA improved SROCC and PLCC by 0.54% and 0.11% in the horizontal view, and by 0.42% and 0.15% in the oblique view, respectively. This demonstrates that introducing the wavelet branch effectively enhances the model’s capability to capture discriminative frequency-related information. In addition, the *p*-values in Table 5 provide further statistical evidence that the wavelet branch and its DWT-based design contribute to the effectiveness of the proposed method.

Moreover, replacing the DWT operation in the wavelet branch with DFT while keeping the remaining branch structure unchanged resulted in consistently worse performance in both the horizontal and oblique views. Compared with Mamba (w/ DFT), WaveMamba-IQA improved SROCC and PLCC by 0.34% and 0.40% in the horizontal view, and by 0.61% and 0.72% in the oblique view, respectively. These results indicate that the superiority of WaveMamba-IQA does not merely come from introducing a frequency-processing branch, but also from the specific use of DWT within that branch. A possible reason is that DWT provides better multi-scale spatial-frequency localization, which is more effective for characterizing local high-frequency details and structural distortions in autofocus images than directly replacing it with DFT. Therefore, the wavelet branch is not only effective, but its DWT-based design is also more suitable for the proposed IQA task.

### 4.3. Autofocus Pipeline Testing

To verify the effectiveness of the proposed method in actual autofocus tasks, this paper evaluated the complete autofocus pipeline under different lighting conditions, conducting a total of 60 independent tests. Each experiment started from a random initial defocus position, using the WaveMamba-IQA sharpness evaluation model to guide the camera through the autofocus process under varying lighting conditions.

Considering that the practical application goal of this study is to obtain clear microsphere contours in the oblique camera view, the best clear position adjusted manually was used as the reference. An autofocus attempt was judged as successful when the axial distance between the final search position of the autofocus and the manual clear position did not exceed 30 µm. This threshold was chosen because contour differences are difficult to distinguish by the human eye within 30 µm.

To set the local-search parameters in Equation (9), *K* was fixed empirically based on repeated trials. Specifically, extensive preliminary experiments confirmed that the sharpest image was always contained within a ±125 µm neighborhood around the estimated position. Over 50 independent trials, the absolute deviation between the estimated oblique clear position and the manually selected clear position had a maximum of 97.2 µm and an average of 71.4 µm, which motivates using ±125 µm as a conservative yet bounded local search range. Accordingly, we set the step size to Δw = 25 µm to balance positioning precision and time cost.

The experimental results are shown in Table 6. Here, Avg Steps (Horizontal) and Avg Steps (Oblique) denote the average focus-search steps for the horizontal and oblique cameras, respectively. Avg Single Inference Time denotes the WaveMamba-IQA inference time for a single step, Avg Total Inference Time denotes the accumulated WaveMamba-IQA inference time per autofocus trial, and Avg Total Time Including Motion denotes the end-to-end runtime including camera motion. Under different lighting conditions, the proposed method was able to stably complete the focusing process with a limited number of focusing steps and short inference time, meeting the operational efficiency requirements of the actual system. In 60 tests, the autofocus success rate of WaveMamba-IQA reached 98.33%, indicating that this method has good stability and robustness in real microscopic focusing scenarios.

## 5. Discussion

The experimental results demonstrate that WaveMamba-IQA is effective for microscopic autofocus quality assessment. As shown in Table 2, the proposed method achieved the best SROCC and PLCC values on both horizontal and oblique views compared with representative NR-IQA methods, indicating more reliable sharpness ranking and quality prediction under dual-view microscopic imaging conditions. The ablation results in Table 4 further show that both the wavelet branch and the DWT-based design contribute to the final performance, confirming the importance of multi-scale frequency information for autofocus image quality evaluation.

From the application perspective, the proposed method also showed strong practical potential in real microscopic autofocus experiments. As reported in Table 6, it achieved a success rate of 98.33% with limited search steps and acceptable execution time, demonstrating its effectiveness for improving autofocus reliability in microsphere micro-assembly. However, the current method still has room for improvement in end-to-end efficiency and in generalization to more complex micro-objects under challenging imaging conditions. In particular, this paper did not evaluate the proposed method on more complex components or in more diverse micro-assembly environments, which should be further investigated in future work.

## 6. Conclusions

This paper presented an efficient two-stage autofocus framework for microsphere micro-assembly based on WaveMamba-IQA. By jointly exploiting spatial and frequency-domain information, the proposed method provides a more effective solution for microscopic image sharpness assessment and autofocus decision-making. The framework combines image quality evaluation with geometric prior guidance, making it suitable for accurate and practical autofocus in micro-assembly scenarios. Although the proposed method has shown good applicability in the current task, its real-time performance and generalization ability for more diverse micro-components still need further improvement. Future work will focus on lightweight model design, acceleration of the end-to-end autofocus process, and extension of the proposed framework to more complex microscopic objects and broader micro-assembly applications.

## Figures and Tables

**Table 1:** Statistical summary of the microsphere dataset.

Dataset	Image Size	No. of Images	No. of Sequences	Images per Sequence
Horizontal Camera	512×512	1600	4	400
Oblique Camera	512×512	800	4	200

**Table 2:** Comparison of WaveMamba-IQA and representative NR-IQA methods, including MANIQA and HyperIQA. Bold entries indicate the best performance.

Method	SROCC (Horizontal)	PLCC (Horizontal)	SROCC (Oblique)	PLCC (Oblique)
MANIQA [18]	0.9704 ± 0.0032	0.9589 ± 0.0041	0.9523 ± 0.0038	0.9410 ± 0.0030
HyperIQA [19]	0.9652 ± 0.0044	0.9511 ± 0.0037	0.9472 ± 0.0031	0.9323 ± 0.0038
WaveMamba-IQA (Ours)	**0.9786** ± 0.0029	**0.9624** ± 0.0023	**0.9598** ± 0.0025	**0.9443** ± 0.0031

**Table 3:** *p*-values of paired two-sided *t*-tests between WaveMamba-IQA and competing methods. Bold entries indicate statistically significant differences (p<0.05).

Comparison	SROCC (Horizontal)	PLCC (Horizontal)	SROCC (Oblique)	PLCC (Oblique)
WaveMamba-IQA vs. MANIQA	**0.0169**	0.1385	**0.0182**	**0.0418**
WaveMamba-IQA vs. HyperIQA	**0.0078**	**0.0001**	**0.0007**	**0.0087**

**Table 4:** Ablation study investigating the effectiveness of the wavelet branch and the choice of frequency decomposition strategy. Bold entries indicate the best performance.

Method	SROCC (Horizontal)	PLCC (Horizontal)	SROCC (Oblique)	PLCC (Oblique)
Mamba (w/o Wavelet)	0.9732 ± 0.0031	0.9613 ± 0.0032	0.9556 ± 0.0028	0.9428 ± 0.0021
Mamba (w/ DFT)	0.9752 ± 0.0033	0.9584 ± 0.0028	0.9537 ± 0.0033	0.9371 ± 0.0037
WaveMamba-IQA	**0.9786** ± 0.0029	**0.9624** ± 0.0023	**0.9598** ± 0.0025	**0.9443** ± 0.0031

**Table 5:** *p*-values of paired two-sided *t*-tests for the ablation study. Bold entries indicate statistically significant differences (p<0.05).

Comparison	SROCC (Horizontal)	PLCC (Horizontal)	SROCC (Oblique)	PLCC (Oblique)
WaveMamba-IQA vs. Mamba (w/o Wavelet)	**0.0473**	0.0524	**0.0280**	**0.0284**
WaveMamba-IQA vs. Mamba (w/ DFT)	**0.0355**	**0.0057**	**0.0101**	**0.0196**

**Table 6:** Autofocus performance evaluation of WaveMamba-IQA in real microscopic scenarios.

Avg Steps (Horizontal)	Avg Steps (Oblique)	Avg Single Inference Time	Avg Total Inference Time	Avg Total Time Including Motion	Success Rate
28	10	0.12 s	4.18 s	18.23 s	98.33%

## Data Availability

The data presented in this study are available on request from the corresponding author due to privacy restrictions.

## References

[1] Zhang J., Dai X., Wu W., Du K. (2023). Micro-Vision Based High-Precision Space Assembly Approach for Trans-Scale Micro-Device: The CFTA Example. Sensors.

[2] Bettahar H., Clevy C., Courjal N., Lutz P. (2020). Force-Position Photo-Robotic Approach for the High-Accurate Micro-Assembly of Photonic Devices. IEEE Robot. Autom. Lett..

[3] Zhang Z., Wang X., Zhao H., Ren T., Xu Z., Luo Y. (2020). The Machine Vision Measurement Module of the Modularized Flexible Precision Assembly Station for Assembly of Micro- and Meso-Sized Parts. Micromachines.

[4] Ruggeri S., Fontana G., Fassi I. (2017). Micro-Assembly. Springer Tracts in Mechanical Engineering.

[5] Shen F., Zhang Z., Xu D., Zhang J., Wu W. (2019). An Automatic Assembly Control Method for Peg and Hole Based on Multidimensional Micro Forces and Torques. Int. J. Precis. Eng. Manuf..

[6] Tamadazte B., Arnould T., Dembele S., Fort-Piat N.L., Marchand E. (2009). Real-time vision-based microassembly of 3D MEMS. 2009 IEEE/ASME International Conference on Advanced Intelligent Mechatronics.

[7] Gibson I., Osterlund E., Truant R. (2025). Using beads as a focus fiduciary to aid software-based autofocus accuracy in microscopy. Bio-Protocol.

[8] Duceux G., Tamadazte B., Le-Fort Piat N., Dembele S., Marchand E., Fortier G. (2010). Autofocusing-Based Visual Servoing: Application to MEMS Micromanipulation. Proceedings of the International Symposium on Optomechatronic Technologies (ISOT).

[9] Subbarao M., Tyan J.-K. (1998). Selecting the optimal focus measure for autofocusing and depth-from-focus. IEEE Trans. Pattern Anal. Mach. Intell..

[10] Pertuz S., Puig D., Garcia M.A. (2013). Analysis of Focus Measure Operators for Shape-From-Focus. Pattern Recognit..

[11] Qu J.W., Xu D., Zhang D.P., Xu J.Z. (2021). High-Precision Measurement Method for Microsphere Hole Pose Based on Active Motion of Two Microscopic Cameras. Acta Autom. Sin..

[12] Her L., Yang X. (2019). Research of Image Sharpness Assessment Algorithm for Autofocus. 2019 IEEE 4th International Conference on Image, Vision and Computing (ICIVC).

[13] Pauwelyn A., Carré M., Jourlin M., Ginhac D., Meriaudeau F. (2025). Image Visual Quality: Sharpness Evaluation in the Logarithmic Image Processing Framework. Big Data Cogn. Comput..

[14] Yu S., Chen Z., Yang Z., Gu J., Feng B. (2024). Exploring Kolmogorov-Arnold networks for realistic image sharpness assessment. arXiv.

[15] Jamil S. (2024). Review of image quality assessment methods for compressed images. J. Imaging.

[16] Herath H.M.S.S., Herath H.M.K.K.M.B., Madusanka N., Lee B.-I. (2025). A systematic review of medical image quality assessment. J. Imaging.

[17] Mao Q., Liu S., Li Q., Jeon G., Kim H., Camacho D. (2025). No-Reference Image Quality Assessment: Past, Present, and Future. Expert Syst..

[18] Yang S., Wu T., Shi S., Lao S., Gong Y., Cao M., Wang J., Yang Y. (2022). MANIQA: Multi-dimension Attention Network for No-Reference Image Quality Assessment. arXiv.

[19] Su S., Yan Q., Zhu Y., Zhang C., Ge X., Sun J., Zhang Y. Blindly Assess Image Quality in the Wild Guided by a Self-Adaptive Hyper Network. Proceedings of the IEEE/CVF Conference on Computer Vision and Pattern Recognition.

[20] Zhu H., Li L., Wu J., Dong W., Shi G. (2020). MetaIQA: Deep Meta-learning for No-Reference Image Quality Assessment. arXiv.

[21] Shi J., Gao P., Qin J. (2024). Transformer-based no-reference image quality assessment via supervised contrastive learning. Proc. AAAI Conf. Artif. Intell..

[22] Yu X., Yu R., Yang J., Duan X. A robotic auto-focus system based on deep reinforcement learning. Proceedings of the 5th International Conference on Control, Automation, Robotics and Vision (ICARCV).

[23] Guan F., Li X., Yu Z., Lu Y., Chen Z. (2024). QMamba: On first exploration of vision mamba for image quality assessment. arXiv.

[24] Wei Y., Liu B., Zhu Z., Ma Y., Liang F., Li Z. (2025). MCN: A mixture capsule network for authentic blind image quality assessment. Knowl. Based Syst..

[25] Lu Y., Li W., Ning X., Dong X., Zhang Y., Sun L. Image quality assessment based on dual domains fusion. Proceedings of the 2020 International Conference on High Performance Big Data and Intelligent Systems (HPBD&IS).

[26] Mallat S.G.A. (1989). Theory for Multiresolution Signal Decomposition: The Wavelet Representation. IEEE Trans. Pattern Anal. Mach. Intell..

[27] Dosovitskiy A., Beyer L., Kolesnikov A., Weissenborn D., Zhai X., Unterthiner T., Dehghani M., Minderer M., Heigold G., Gelly S. (2020). An Image is Worth 16x16 Words: Transformers for Image Recognition at Scale. arXiv.

[28] Zamir S.W., Arora A., Khan S., Hayat M., Khan F.S., Yang M.-H. Restormer: Efficient Transformer for High-Resolution Image Restoration. Proceedings of the IEEE/CVF Conference on Computer Vision and Pattern Recognition.

[29] Zhu L., Liao B., Zhang Q., Wang X., Liu W., Wang X. (2024). Vision Mamba: Efficient Visual Representation Learning with Bidirectional State Space Model. arXiv.

[30] Hansen N. (2016). The CMA Evolution Strategy: A Tutorial. arXiv.

[31] Xu K., Qin M., Sun F., Wang Y., Chen Y.-K., Ren F. (2020). Learning in the Frequency Domain. arXiv.

[32] Gu A., Dao T. (2023). Mamba: Linear-Time Sequence Modeling with Selective State Spaces. arXiv.

[33] Briggs W.L., Henson V.E. (1995). The DFT: An Owner’s Manual for the Discrete Fourier Transform.

